# Supplementary material for: Statins to reduce renal sinus fat among breast cancer patients undergoing anthracycline-based chemotherapy: A substudy of PREVENT-WF-98213
Source: PLoS One. 2025 Sep 16;20(9):e0318017. doi: 10.1371/journal.pone.0318017 (PMC12440191; doi:10.1371/journal.pone.0318017)
Supplement: S1 Table — (DOC) [file pone.0318017.s001.doc]

| **Supplemental Table 1.** Comparison of baseline demographic and clinical characteristics of women with breast cancer in the parent study and current study | | | |
| --- | --- | --- | --- |
| **Characteristic** | **Excluded in current study**  **N=200** | **Included in current study**  **n=79** | **p-value** |
| **Age (years)** | 48.1 ± 12.3 | 51.3 ± 10.9 | 0.03 |
| **Race** |  |  | 0.22 |
| **White** | 69 (87) | 69 (87) |  |
| **Black** | 28 (10) | 10 (13) |  |
| **Asian** | 3 (1) | 0 (0) |  |
| **Native Hawaiian** | 4 (1) | 0 (0) |  |
| **Native American/Alaskan** | 2 (<1) | 0 (0) |  |
| **Unknown** | 1 (<1) | 0 (0) |  |
| **Female sex** | 177 (89) | 79 (100) | <0.001 |
| **Study group** |  |  | 0.22 |
| **Statin** | 95 (48) | 44 (56) |  |
| **Body mass index (kg/m^2^)** | 29.9 ± 7.3 | 30.2 ± 6.1 | 0.75 |
| **Hypertension medications** | 16 (21) | 16 (21) | 0.70 |
| **Cancer type** |  |  |  |
| **Breast** | 159 (80) | 79 (100) | <0.001 |
| **Lymphoma** | 41 (20) | 0 (0) |  |
| **Cancer stage** |  |  | 0.06 |
| **I** | 38 (19) | 8 (10) |  |
| **II** | 107 (53) | 47 (60) |  |
| **III** | 50 (25) | 24 (30) |  |
| **IV** | 5 (3) | 0 (0) |  |
| **Cumulative anthracycline dose (mg/m^2^)** | 232.8 ± 60.0 | 236.5 ± 26.7 | 0.49 |
| Data are presented as mean ± SD or n (%).  N missing for hypertension medications in subset cohort=2; n missing in total cohort n=5  N missing for cumulative anthracycline dose in subset cohort=3; n missing in total cohort n=22 | | | |
