# Supplementary material for: Statins to reduce renal sinus fat among breast cancer patients undergoing anthracycline-based chemotherapy: A substudy of PREVENT-WF-98213
Source: PLoS One. 2025 Sep 16;20(9):e0318017. doi: 10.1371/journal.pone.0318017 (PMC12440191; doi:10.1371/journal.pone.0318017)
Supplement: S2 Table — (DOC) [file pone.0318017.s002.doc]

| **Supplemental Table 2.** Regression models of inflammatory markers (predictor) and renal sinus fat (outcome). | | | |
| --- | --- | --- | --- |
| **Term** | **β ± standard error** | **95% CI** | **p-value** |
| Intercept | 0.28 ± 0.21 | -0.16, 0.71 | 0.20 |
| Change in C-reactive protein (CRP)* | 0.003 ± 0.02 | -0.03, 0.04 | 0.85 |
| Baseline renal sinus fat | 0.77 ± 0.14 | 0.48, 1.06 | <0.001 |
|  | | | |
|  | **β ± standard error** | **95% CI** | **p-value** |
| Intercept | 0.29 ± 0.21 | -0.12, 0.71 | 0.16 |
| Change in tumor necrosis factor-α (TNF-α) | -0.06 ± 0.04 | -0.15, 0.03 | 0.17 |
| Baseline renal sinus fat | 0.75 ± 0.14 | 0.47, 1.03 | <0.001 |
|  |  |  |  |
|  | **β ± standard error** | **95% CI** | **p-value** |
| Intercept | 0.29 ± 0.22 | -0.16, 0.73 | 0.20 |
| Change in interleukin-6 (IL-6) | -0.01 ± 0.05 | -0.11, 0.09 | 0.81 |
| Baseline renal sinus fat | 0.76 ± 0.14 | 0.47, 1.05 | <0.001 |
| Change in inflammatory markers was calculated as baseline values subtracted from 24-month values. Renal sinus fat values were square root transformed to account for a non-normal distribution.  N=35 for all analyses | | | |
