# Supplementary material for: Statins to reduce renal sinus fat among breast cancer patients undergoing anthracycline-based chemotherapy: A substudy of PREVENT-WF-98213
Source: PLoS One. 2025 Sep 16;20(9):e0318017. doi: 10.1371/journal.pone.0318017 (PMC12440191; doi:10.1371/journal.pone.0318017)
Supplement: Attachment — Submitted filename: Response to Reviewers.docx [file pone.0318017.s006.docx]

**Reviewer #1:** 

1. This is a very disappointing revision that takes little note of the suggestions I made in my earlier review. Focusing on the Results section of the current Abstract, it begins by presenting differences in patient characteristics in the two groups. The real focus should be on Statin v Placebo and if a difference between these groups is demonstrated, is the size of the effect influenced by the covariates?

**Response:** We apologize for the oversight, and we have now removed comparisons of participant characteristics from the abstract. Rather, we now describe cohort characteristics overall for readers and then present results for statin vs. placebo groups. In addition, we have thoroughly revised our analyses, results, and discussion in line with suggestions in your first review, which we describe in later comments.

Abstract excerpt regarding participant characteristics:

“Participants in this study averaged 50.0 years of age (SD 10.9), 86% reported White race, and had a mean BMI (±SD) of 29.9 kg/m^2^ (±5.9). Most participants (66%) were diagnosed with stage II BC.”

2. My earlier review emphasized the need for confidence intervals. The authors have calculated some, but none of these are included in the Abstract. Thus, the Abstract needs redrafting with a clear focus on Statin v Placebo with respect to RSF.

**Response:** We apologize for the oversight of not including the confidence intervals requested in the abstract. We have now included those confidence intervals for the statistical estimates shown within the abstract and results text. Analyses have been adjusted in line with your suggestions and are described below (comment 7). As you suggested, the abstract results present findings between the statin and placebo groups. As one aim of the paper was to test for associations between RSF and kidney function, we do include one correlation within all participants at baseline (this study visit took place prior to beginning cancer treatment and study group allocation) and then correlation analyses are presented stratified by study group. Otherwise, all results in the abstract are focused on differences in the placebo and statin groups.

3. The Discussion section is very weak indeed.

**Response:** We have revised the discussion in line with updated analyses and conclusions from this major revision.

4. This (subgroup) fact needs more prominence within the title of the paper and in the Abstract.

**Response:** We have amended our title to include that this is a subgroup analysis. We have also included in the abstract text stating “this was a *secondary analysis* in a *subgroup* of women…”. In addition, this is stated in the introduction and methods sections, and there is a flow chart showing the subgroup included in this analysis. We also emphasize in the discussion that this is a small sample of the parent trial.

New title: “Statins to reduce renal sinus fat among breast cancer patients undergoing anthracycline-based chemotherapy: A substudy of PREVENT-WF-98213”

5. Further the r2 values provide no useful information and can also be omitted.

**Response:** We have now removed the r^2^ values.

6. In general, it is of no value to make statistical comparisons of the covariates (say between age groups) as their effect on any difference between the two design groups is taken account of in the modelling process.

**Response:** We apologize for the oversight in removing statistical comparisons in the abstract. We have now removed those comparisons from the abstract body. To our knowledge, there are now no other statistical comparisons of covariates. In the results section, we describe characteristics of the cohort but do not make comparisons. Text within the results and p-values comparing covariates were removed from the results and Table 1 in the first revision.

7. Setting a fixed value for significance is not a good idea neither is it likely that the modelling approach with an Interaction term included is justified.

**Response:** Our initial analyses included an intermediate timepoint, which was removed due to less data availability. Those original analyses were preserved; however; we thank the reviewer because we now understand that the mixed model and interaction term included was not appropriate for the current analysis with two timepoints in this study design.

We have now updated our analyses to match those that you suggested in the first revision. That is, first we compare renal sinus fat levels at 24-months between placebo and statin groups (model 1 shown below). We then subsequently perform a linear regression analysis with renal sinus fat at 24-months as the outcome and study group (placebo or statin) as the main predictor while also adjusting for baseline renal sinus fat (model 2 results below). From these results, we find a difference in renal sinus fat levels at 24-months between groups in model 1. In the second model after adjusting for baseline levels, this signal remains but was attenuated. While the effect size is smaller than our original findings, given that this signal remains with a modest sample size, we believe this may warrant future considerations if statins impact renal sinus fat levels.

Our revised analyses are presented in Table 3 and described in the results section.

| **Table 3.** Analyses comparing renal sinus fat at 24-months between the placebo and statin groups | | | |
| --- | --- | --- | --- |
| **Model 1 Outcome: 24 month renal sinus fat** | | | |
|  | **β ± standard error** | **95% CI** | **p-value** |
| **Intercept** | 1.25 ± 0.08 | 1.08, 1.41 | <0.001 |
| **Placebo group** | 0.17 ± 0.08 | 0.009, 0.34 | 0.04 |
|  | | | |
| **Model 2 Outcome: 24 month renal sinus fat** | | | |
|  | **β ± standard error** | **95% CI** | **p-value** |
| **Intercept** | 0.33 ± 0.21 | -0.09, 0.76 | 0.12 |
| **Placebo group** | 0.12 ± 0.08 | -0.06, 0.29 | 0.18 |
| **Baseline renal sinus fat** | 0.73 ± 0.14 | 0.46, 1.01 | <0.001 |
| Renal sinus fat values were square root transformed to account for a non-normal distribution. The first model shows results from a Student’s t-test comparing 24-month renal sinus fat between groups (n=54). The second model shows results from a linear regression model comparing 24-month renal sinus fat between groups while adjusting for baseline renal sinus fat (n=35). | | | |

**Reviewer #4:**

1. Methods/Line 146-148: “Additional covariates considered but not included as they were not associated with renal sinus fat were cancer stage, education level, marital status, income, smoking status, and cumulative anthracycline dose.” Please indicate why you have not applied a multivariate analysis, since your study group is relatively small (~79 patients) and you need to assess parameters that associate with breast cancer, and HMG-CoA reductase inhibitors. You are including these to avoid confounding effects or biases due to small study size since your study size is not enough to provide sufficient randomization. Please also specify if you have conducted a linear/log/Poisson regression or cox survival analysis.

**Response:** We apologize for any confusion regarding the analyses. Initially, we did apply multivariate analyses in this project. However, we thank you for these comments and those from Reviewer 1 because we now recognize that our initial analyses were inappropriate for using two timepoints with the current study design. In line with those comments and suggestions, we now present results comparing renal sinus fat at 24-months between groups. We then present a model comparing renal sinus fat at 24-months between study groups while adjusting for baseline renal sinus fat. As you have mentioned, the sample size is very limited (n=35 total in model 2 below), so we did not include additional covariates. These results are shown in Table 3.

| **Table 3.** Analyses comparing renal sinus fat at 24-months between the placebo and statin groups | | | |
| --- | --- | --- | --- |
| **Model 1 Outcome: 24 month renal sinus fat** | | | |
|  | **β ± standard error** | **95% CI** | **p-value** |
| **Intercept** | 1.25 ± 0.08 | 1.08, 1.41 | <0.001 |
| **Placebo group** | 0.17 ± 0.08 | 0.009, 0.34 | 0.04 |
|  | | | |
| **Model 2 Outcome: 24 month renal sinus fat** | | | |
|  | **β ± standard error** | **95% CI** | **p-value** |
| **Intercept** | 0.33 ± 0.21 | -0.09, 0.76 | 0.12 |
| **Placebo group** | 0.12 ± 0.08 | -0.06, 0.29 | 0.18 |
| **Baseline renal sinus fat** | 0.73 ± 0.14 | 0.46, 1.01 | <0.001 |
| Renal sinus fat values were square root transformed to account for a non-normal distribution. The first model shows results from a Student’s t-test comparing 24-month renal sinus fat between groups (n=54). The second model shows results from a linear regression model comparing 24-month renal sinus fat between groups while adjusting for baseline renal sinus fat (n=35). | | | |

2. Methods/ Line 154: please reason why 10% statistical significance was chosen. Please also cite suitable references in the same field that mention they have used the same values.

**Response:** Thank you for this question. Initially, a p<0.1 was selected only for interaction terms to remain in models. However, from your comment and Reviewer 1’s comments, we recognize that our analyses should not have included interactions terms, and we have now adjusted our analyses and results as suggested above.

3. Methods: please provide sample size calculations, and cite relevant articles.

**Response:** When comparing groups, there was a marginally significant finding at 24-months suggesting that the statin group had better levels of renal sinus fat (RSF) compared to the placebo group (p=0.04); however, we recognized these groups were not identical to baseline groups, so we subsequently adjusted for baseline RSF values. When accounting for this, we found that our signal was similar but attenuated (p=0.04 became p=0.18) and this was because 1) placebo group RSF levels were slightly higher at baseline (0.2 units higher) and 2) sample size was greatly reduced (n=35). However, the signal still suggests that after adjustment, the groups are 0.23 units apart (mean difference). With 95 participants per group, we would have 80% power to detect a 0.23 unit mean difference. This sample size would have been possible in the larger Prevent study cohort (N=279), but our analyses were conducted on a subsample of the Prevent study with available RSF data resulting in a much smaller sample size.

With our baseline sample of 79 participants, we had sufficient sample size to detect a clinically meaningful correlation; however, with 24-month comparisons adjusting for baseline data, we did not see a statistically significant effect between groups due to sample size although the signal for a difference remained. Had we observed the mean difference reported here with 100 participants per group, we would have found a statistically significant finding. We have now added this information to our discussion.

4. Tables: please change ß to OR, RR, or HR depending on your analysis. Please name the exact analysis that you have used.

**Response:** In response to other reviewer comments, we have changed our analyses. We now use linear regression analyses and present beta estimates, confidence intervals, and p-values for those results. In these analyses comparing renal sinus fat, the β estimate corresponds to the mean difference in renal sinus fat on a square root scale between study groups. We have now updated our statistical analysis section, results, and tables to reflect these changes.

5. Please also justify why you have reported serum creatinine along with eGFR, and of what excess value does serum creatinine provides.

**Response:** We agree with you that including serum creatinine does not add meaningful information as the results mirror those of eGFR. We have included the mean concentrations of serum creatinine as a descriptive clinical assessment in Table 2, but we have now removed results pertaining to serum creatinine and focus only on eGFR.
